# Effect of severe acute respiratory syndrome coronavirus 2 infection during pregnancy in K18-hACE2 transgenic mice

**DOI:** 10.5713/ab.22.0143

**Published:** 2022-09-02

**Authors:** Byeongseok Kim, Ki Hoon Park, Ok-Hee Lee, Giwan Lee, Hyukjung Kim, Siyoung Lee, Semi Hwang, Young Bong Kim, Youngsok Choi

**Affiliations:** 1 Department of Stem Cell and Regenerative Biotechnology, Konkuk University, Seoul 05029, Korea; 2 Department of Research and Development, KR BIOTECH CO., Ltd., Seoul, 05029, Korea; 3 Department of Veterinary Physiology, College of Veterinary Medicine, Konkuk University, Seoul, 05029, Korea; 4 Department of Biomedical Science and Engineering, Konkuk University, Seoul, 05029, Korea

**Keywords:** Implantation, K18-hACE2 Transgenic Mouse, Pregnancy, SARS-CoV-2

## Abstract

**Objective:**

This study aimed to examine the influence of severe acute respiratory syndrome coronavirus 2 (SARS-CoV-2) infection on pregnancy in cytokeratin-18 (K18)-hACE2 transgenic mice.

**Methods:**

To determine the expression of hACE2 mRNA in the female reproductive tract of K18-hACE2 mice, real-time polymerase chain reaction (RT-PCR) was performed using the ovary, oviduct, uterus, umbilical cord, and placenta. SARS-CoV-2 was inoculated intranasally (30 μL/mouse, 1×10^4^ TCID_50_/mL) to plug-checked K18-hACE2 homozygous female mice at the pre-and post-implantation stages at 2.5 days post-coitum (dpc) and 15.5 dpc, respectively. The number of implantation sites was checked at 7.5 dpc, and the number of normally born pups was investigated at 20.5 dpc. Pregnancy outcomes, including implantation and childbirth, were confirmed by comparison with the non-infected group. Tissues of infected mice were collected at 7.5 dpc and 19.5 dpc to confirm the SARS-CoV-2 infection. The infection was identified by performing RT-PCR on the infected tissues and comparing them to the non-infected tissues.

**Results:**

hACE2 mRNA expression was confirmed in the female reproductive tract of the K18-hACE2 mice. Compared to the non-infected group, no significant difference in the number of implantation sites or normally born pups was found in the infected group. SARS-CoV-2 infection was detected in the lungs but not in the female reproductive system of infected K18-hACE2 mice.

**Conclusion:**

In K18-hACE2 mice, intranasal infection with SARS-CoV-2 did not induce implantation failure, preterm labor, or miscarriage. Although the viral infection was not detected in the uterus, placenta, or fetus, the infection of the lungs could induce problems in the reproductive system. However, lung infections were not related to pregnancy outcomes.

## INTRODUCTION

The number of confirmed cases of COVID-19 in 191 countries is approximately 458 million, including 6 million deaths by March 2022 [1]. The COVID-19 pandemic resulted in maternal deaths and stillbirths; thus, pregnant women and newborns are at a high risk of being affected by COVID-19 [2]. Therefore, there is a demand for research on the effects of COVID-19 on pregnancy, childbirth, and fetal growth.

The representative pathogen of the COVID-19 pandemic, severe acute respiratory syndrome coronavirus 2 (SARS-CoV-2), causes symptoms of fever, cough, and dyspnea at an early infection stage [3,4]. Severe symptoms of COVID-19 are accompanied by pulmonary edema, bilateral diffuse alveolar damage, and hyaline membrane formation [3,4]. The spike glycoprotein of SARS-CoV-2 penetrates cells through interaction with angiotensin-converting enzyme 2 (ACE2) [5]. In humans, ACE2 is expressed in the central nervous system, cardiovascular system, kidneys, gut, and adipose tissues and is highly expressed in the lung epithelium [6]. Notably, ACE2 expression was found in human reproductive systems. To date, studies have confirmed its expression in the ovary, uterus, umbilical cord, and placenta [7]. Therefore, there is a possibility of SARS-CoV-2 infection of women's reproductive systems.

Most pathogenic viruses that induce infection cannot reach the fetus through the placenta, especially because of syncytiotrophoblasts, the placental barrier between the maternal and fetal blood [8–10]. However, studies have shown that viruses such as herpes virus (HSV), cytomegalovirus (CMV), rubella virus, and Zika virus (ZIKV) can pass through the placenta and infect the fetus [11,12]. In addition, in the case of ZIKV, the damage to the placenta negatively induces fetal demise and affects neuronal development [13,14]. Several studies have found that SARS-CoV-2 can also infect the fetus through the placenta. In 2020, COVID virions were found in the placentas of infected patients, and the RNA of SARS-CoV-2 and immunoglobulin M were also found in newborn infants, according to several studies [15–17]. Recently, there have been reports that SARS-CoV-2 was discovered in amniotic fluid and umbilical blood [18]. Considering these results, there is a possibility of vertical transmission of SARS-CoV-2 through the placental barrier, but it is still unclear.

To determine the effect of SARS-CoV-2 on the reproductive system of pregnant women, we used a cytokeratin-18 (K18)-hACE2 transgenic mouse model, in which hACE2 expression is driven by the K18 promoter [19]. Herein, we demonstrated the effect of SARS-CoV-2 on embryo implantation and development by infecting pregnant mice with SARS-CoV-2 at the pre- and post-implantation stages of K18-hACE2 transgenic mice.

## MATERIALS AND METHODS

### Preparation of the virus

The SARS-CoV-2 Wuhan strain was obtained from the National Culture Collection for Pathogens and amplified in the Vero-E6 cell line (ATCC, CRL-1586). Vero-E6 cells were maintained in Dulbecco's modified eagle medium (Cytiva, Marlborough, MA, USA) containing 10% fetal bovine serum (Invitrogen, Carlsbad, CA, USA) and 1% penicillin/streptomycin (P/S; Gibco, Waltham, MA, USA). And cells were incubated at 37°C with 5% CO_2_. A day before the infection, 8×10^6^ cells of Vero-E6 were seeded into a T75 flask (SPL Life Science, Pocheon, Korea). Before the infection, the medium was removed without disrupting the cell monolayer, and 10 mL of fresh medium was added. Vero-E6 cells were infected with SARS-CoV-2 and after two days of the infection, the culture media were harvested and centrifuged at 3,000 rpm for 10 min to remove cell debris. The harvested viruses were stored at −80°C.

### Viral titration

Viral titration was performed using the 50% tissue culture infectious dose (TCID_50_) method. A day before the infection, 1×10^4^ Vero-E6 cells were seeded into a flat bottom 96-well plate (SPL Life Science, Korea). The viruses were 10-fold serially diluted and used to infect Vero-E6 cells as 100 μL per well. After three days of incubation, the cells were stained with a crystal violet solution to evaluate the cytopathic effect [20]. The TCID_50_ was determined using the Spearman–Karber method [21,22].

### Animal experiments

The K18-hACE2 heterozygous transgenic mice were provided by Jackson Laboratories (USA) and the wild-type C57BL/6J mice by JA Bio (Suwon, Korea). The mice were kept under standard conditions (23°C±1°C and 50%±2% humidity) in a light:dark (12 h:12 h) cycle. Animals were allowed free access to water and food. All experiments were performed according to the institutional guidelines approved by the Konkuk University Institutional Animal Care and Use Committee (approval no. KU20072). Genotyping of K18-hACE2 homozygous mice was carried out by performing polymerase chain reaction (PCR) on genomic DNA, according to the manufacturer's instructions. Briefly, the end part of mice tail was used for genotyping and PCR was conducted with extracted gDNA. With the PCR product, incubation of restriction enzymes with HpyCH4V (NEB, Ipswich, MA, USA) was followed. In case of heterozygous mice, three products of 285, 148, and 137 bp are present. Just one product of 285 bp is confirmed in the wildtype mice and two products of 148 and 137 bp are identified in the homozygous mice. The K18-hACE2 homozygous transgenic mice (n = 5) were intranasally inoculated with SARS-CoV-2 according to three different virus titers (1×10^2^, 1×10^3^, 1×10^4^ TCID_50_/mouse, 30 μL). Body weights and survival rate of mice were monitored after infection. To confirm the reproductive results of transgenic mice, mating was conducted between each genotype. The mice were set up for timed mating, and copulatory plugs were checked every morning (0.5 days post-coitum [dpc]). The age of used mice was between 7 to 8 weeks (19 to 20 g) for female mice and was over 8 weeks (25 to 29 g) for male mice. K18-hACE2 homozygous mice (n = 3) were inoculated intranasally with SARS-CoV-2 (1×10^4^ TCID_50_/mouse, 30 μL) at 2.5 and 15.5 dpc and sacrificed at 7.5 and 19.5 dpc depending on the experimental design. The inoculation was conducted with three independent experiments. Implantation sites were checked in 7.5 dpc mice, and the number of normally born pups was checked at 20.5 dpc. The ovary, uterus, and lungs were sampled from 7.5 dpc mice. The amnion, fetus, placenta, and umbilical cord were collected at 19.5 dpc through cesarean section. The collected tissues were stored in 600 μL of RNAlater (Thermo Fisher Scientific, Waltham, MA, USA) until RNA extraction and the lungs were stored in 4% paraformaldehyde (PFA) until hematoxylin and eosin staining. The virus challenge was conducted in a biosafety level 3 animal facility at Konkuk University.

### RNA extraction and reverse transcription-polymerase chain reaction

The tissues stored in RNAlater were placed in a 2-mL tube with RLT buffers, including 1% β-mercaptoethanol and 5-mm stainless steel beads (Qiagen, Hilden, Germany). Tissues were homogenized using a Qiagen TissueLyser II (Qiagen, Germany). After the centrifugation of the homogenized samples, the supernatant was used to isolate the total RNA. According to the manufacturer's instructions, total RNA was extracted using an RNeasy Mini Kit (Qiagen, Germany). To remove the genomic DNA, an additional on-column DNase treatment (Qiagen, Germany) was used. The quality and quantity of total RNA were estimated using a NanoDrop 2000 spectrophotometer (Thermo Fisher Scientific, USA), and cDNA was synthesized from 1 μg of RNA using a SensiFASTcDNA Synthesis Kit (Biloine, Taunton, MA, USA). Synthesized cDNA was diluted to concentration of 5 ng/μL. Real-time PCR (RT-PCR) was conducted with 5 ng of cDNA using Solg Taq DNA polymerase (SolGent, Daejeon, Korea) and each primer to amplify the target genes. The list of primers and annealing temperature is presented in Table 1. Denaturation phase was performed in 95°C, 1 min and the extension phase in 72°C, 15 s. The products of RT-PCR were analyzed in 2% agarose gel. The list of primers used to amplify the target gene is presented in Table 1.

### Hematoxylin and eosin staining

Lungs stored in 4% PF were embedded in paraffin and sectioned to a thickness of 5 μm using microtome. After deparaffinization with standard procedures, lung tissue was stained with hematoxylin (Vector laboratory, Burlingame, CA, USA) and eosin Y (Sigma-Aldrich, St. Louis, MO, USA) and examined using an optical microscope.

### Statistical analyses

All experimental data are expressed as the standard error of the mean (±SEM). Student’s t-test and one-way analysis of variance was used to statistically evaluate the results. Statistical significance was set at p-value <0.05.

## RESULTS

### Expression of hACE2 mRNA in the reproductive system of K18-hACE2 transgenic mice

**Figure 1:**
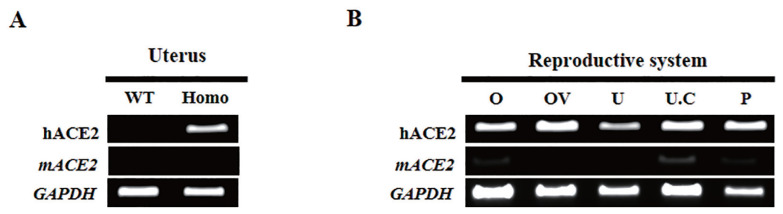
(A) RT-PCR showing hACE2 expression in the uterus of K18-hACE2 transgenic mice compared to non-transgenic mice. RT-PCR, real-time polymerase chain reaction; WT, C57BL/6 mice; Homo, K18-hACE2 homozygous transgenic mice. (B) RT-PCR showing hACE2 expression in the reproductive system of K18-hACE2 transgenic homozygous mice. O, ovary; OV, oviduct; U, uterus; U.C, umbilical cord; P, placenta.

Recent studies have confirmed the expression of ACE2 in the human reproductive system [23]. The expression of ACE2 in the uterus, ovary, and placenta suggests that a SARS-CoV-2 infection may affect embryo implantation and pregnancy. To investigate the effect of SARS-CoV-2 on the female reproductive system, K18-hACE2 homozygous transgenic mice expressing the receptor for viral entry into host cells were used. RT-PCR was performed using the uteri of K18-hACE2 homozygous transgenic female mice and wild-type female mice (C57BL/6J) to confirm the mRNA expression of hACE2. mRNA expression of hACE2 was not detected in the uteri of wild-type mice but in the uteri of K18-hACE2 homozygous mice (Figure 1A). Similar to other studies in which mACE2 expression was not detected in the myometrium of mice [24], mACE2 was not expressed in the uteri of the mice. Additionally, the expression of hACE2 mRNA was detected in the ovary, oviduct, uterus, umbilical cord, and placenta (Figure 1B).

### Effect of SARS-CoV-2 infection according to virus titers

**Figure 2:**
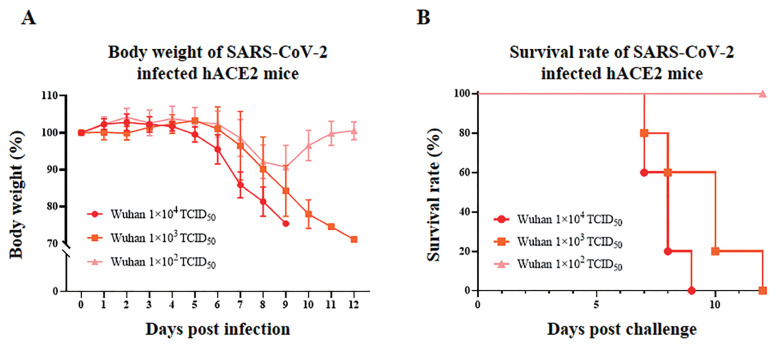
(A) Body weight in SARS-CoV-2 infected K18-hACE2 transgenic homozygous mice (n = 5) according to the virus titers. Results are presented as average change in body weight±standard error of the mean. (B) The mouse survival rate in SARS-CoV-2 infected K18-hACE2 transgenic homozygous mice (n = 5) according to the virus titers. SARS-CoV-2, severe acute respiratory syndrome coronavirus 2; K18, cytokeratin-18.

Before confirming the effect of SARS-CoV-2 on the reproductive system, non-pregnant K18-hACE2 homozygous mice were infected with three different virus titers. In the case of 1×10^2^ TCID_50_, body weights decreased to 90% at 8 dpi but recovered at 12 dpi, and all mice survived until 12 days post-infection (dpi) (Figure 2A, 2B). In the case of 1×10^3^ TCID_50_, the body weights started to decrease at 8 dpi and steadily decreased to 12 dpi, and 20% of mice died at each 7 dpi and 8 dpi, 40% at 10 dpi, and 20% at 12 dpi (Figure 2A, 2B). In the case of 1×10^4^ TCID_50_, the body weights started to gradually decrease from 6dpi and decreased by nearly 80% at 8 dpi, and 40% of mice died at 7 dpi and 8 dpi, respectively, and the rest of mice died at 9 dpi (Figure 2A, 2B). Through these results, the inoculation of pregnant K18-hACE2 homozygous mice was conducted with virus titers, 1×10^4^ TCID_50_/mouse.

### Effect of SARS-CoV-2 infection at the pre-implantation stage

**Figure 3:**
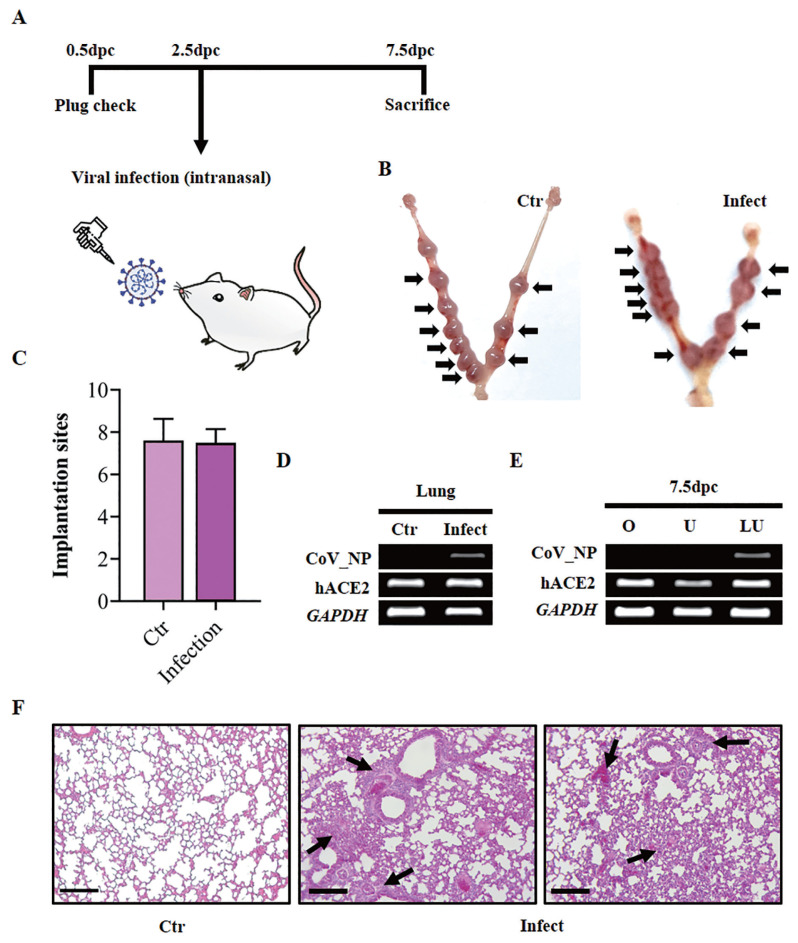
(A) Schematic describing the experiment and plan to examine the effect of SARS-CoV-2 infection on the pre-implantation stage. SARS-CoV-2, severe acute respiratory syndrome coronavirus 2. (B) Representative images showing embryo implantation sites in SARS-CoV-2 infected or uninfected mice at 7.5 days post-coitum (dpc). Black arrows indicate implantation sites. K18, cytokeratin-18. Ctr, uninfected K18-hACE2 mouse uterus; Infection, infected K18-hACE2 mouse uterus. (C) The average number of implantation sites per mouse at 7.5 dpc (n = 9). Data are shown with the mean±standard error of the mean and was analyzed with the student’s t-test. (D) RT-PCR results showing SARS-CoV-2 nucleocapsid RNA expression in the lung from a virus-infected pregnant K18-hACE2 mouse. RT-PCR, real-time polymerase chain reaction; Ctr, uninfected lung of 7.5 dpc K18-hACE2 mouse; Infect, lung of 7.5 dpc K18-hACE2 mouse which was infected with the virus. (E) RT-PCR showing SARS-CoV-2 nucleocapsid RNA expression in a pregnant K18-hACE2 mouse after viral infection. O, ovary; U, uterus; Lu, lung. (F) Assessments of H&E staining in lungs of SARS-CoV-2 infected mice at 7.5 dpc. Scale bar = 100 μm. Black arrow, lesions in the lung; Ctr, uninfected lungs of K18-hACE2 homozygous transgenic mice; Infect, infected lungs of 7.5 dpc K18-hACE2 mice.

To determine the effect of SARS-CoV-2 infection on implantation, K18-hACE2 homozygous female mice were mated with K18-hACE2 homozygous male mice. After the identification of the vaginal plug, intranasal inoculation of the virus was performed during the pre-implantation stage, 2.5 dpc (Figure 3A). Five days after infection, the mice were sacrificed, and viral infection was confirmed by RT-PCR using primers for the sequence of the SARS-CoV-2 nucleocapsid (CoV_NP). Viral nucleocapsid RNA was clearly detected in the lungs of infected K18-hACE2 homozygous mice, confirming a SARS-CoV-2 infection (Figure 3D). No virus was detected in the lungs of non-infected control mice (Figure 3D). Compared to the lungs of non-infected control mice, severe lesions including the decrease of pulmonary alveoli were examined from the lungs of infected mice (Figure 3F). To check the effect of SARS-CoV-2 infection on embryo implantation, the whole uterus was collected, and implantation sites were counted. We discovered that the virus had no significant effect on embryo implantation. The average number of implantation sites in infected mice (n = 9) (8.5 sites per mouse±SEM of 0.5) was similar to that in control mice (n = 9) (9.25 sites per mouse±SEM of 0.41) (Figure 3B, 2C). The uterus and ovaries were collected to determine the penetration of SARS-CoV-2 into the female reproductive system. As shown in Figure 3E, viral nucleocapsid RNA was not detected in the ovaries and uteri of the infected mice, suggesting no viral penetration into the female reproductive system. These data indicated that a SARS-CoV-2 infection does not directly affect embryo implantation.

### Effect of SARS-CoV-2 infection at the post-implantation stage

**Figure 4:**
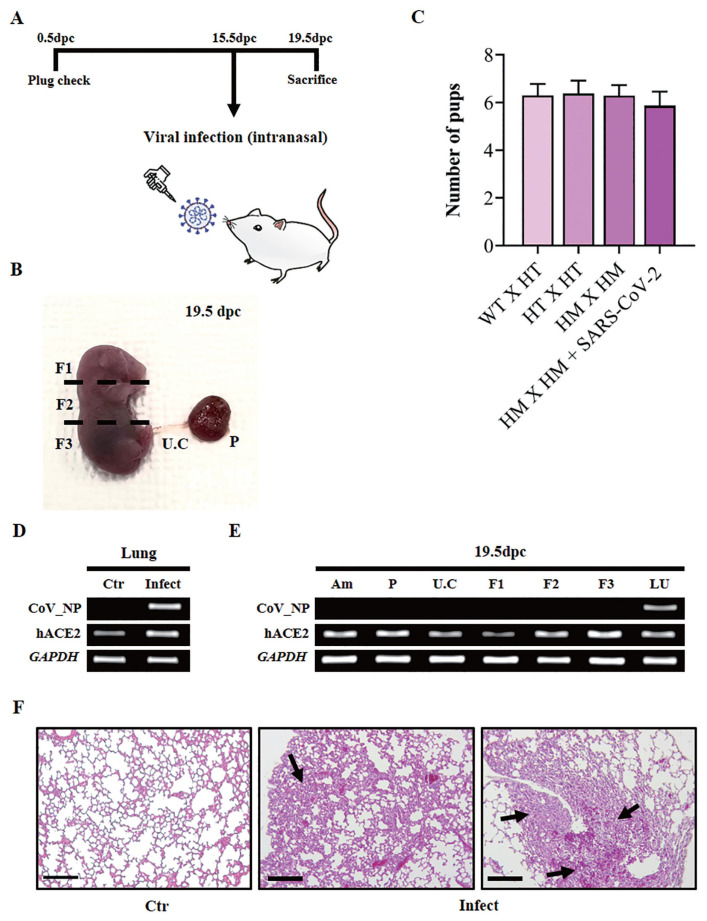
(A) Schematic describing the experiment and plan to examine the effect of SARS-CoV-2 infection on the post-implantation stage. SARS-CoV-2, severe acute respiratory syndrome coronavirus 2. (B) Representative image of 19.5 days post-coitum (dpc) fetus from a non-infected mother. Viral transmission from mother to fetus was determined by RT-PCR using the top (F1), middle (F2), and bottom (F3) parts of the fetus, umbilical cord (U.C), and placenta (P). RT-PCR, real-time polymerase chain reaction. (C) Average number of pups per K18-hACE2 mouse after SARS-CoV-2 infection. Homozygous female mice mated with homozygous male mice were infected with SARS-CoV-2 at 15.5 dpc (n = 9). Data are shown with the mean±standard error of the mean. WT, C57BL/6 mice; K18, cytokeratin-18; HT, heterogenic K18-hACE2 transgenic mice; HM, homozygous K18-hACE2 transgenic mice. (D) RT-PCR showing the expression of SARS-CoV-2 nucleocapsid RNA in the lungs of a pregnant mouse. Control, non-infected lung of 19.5 dpc K18-hACE2 mouse; Covid, lung of 19.5 dpc K18-hACE2 mouse which was infected at 15.5 dpc. (E) RT-PCR results showing SARS-CoV-2 nucleocapsid RNA expression in amnion (Am), placenta (P), umbilical cord (U.C), and fetus (F1, top part; F2, middle part; F3, bottom part) from a virus-infected K18-hACE2 mouse and in the lungs (LU) of a virus-infected pregnant K18-hACE2 mouse. (F) Assessments of H&E staining in lungs of SARS-CoV-2 infected mice at 19.5 dpc. Scale bar = 100 μm. Black arrow, lesions in the lungs; Ctr, uninfected lungs of K18-hACE2 homozygous transgenic mice; Infect, infected lungs of 19.5 dpc K18-hACE2 mice.

To verify the effect of a SARS-CoV-2 infection in the post-implantation stage, viral inoculation was performed at 15.5 dpc (Figure 4A). Uninfected K18-hACE2 homozygous mice were used as controls. The infected mice were sacrificed at 19.5 dpc. The amnion, placenta, umbilical cord, fetus, and lung were collected to confirm the maternal–fetal trans-vertical infection of SARS-CoV-2. The amnion, placenta, umbilical cord, and fetus were collected through cesarean section, and the fetus was divided into three sections (F1, F2, and F3) from the head (Figure 4B). The effect of SARS-CoV-2 infection on the normal birth rate was determined by comparing the number of normally born pups between the infected K18-hACE2 homozygous group and the uninfected group at 15.5 dpc (Figure 4C). Wild-type (WT), K18-hACE2 heterozygous (HT), and K18-hACE2 homozygous (HM) mice were used for mating (Figure 4C). In the uninfected group, the average number of normally born pups was 6.29±SEM of 0.47 (n = 27) for WT×HT, 6.38±SEM of 0.52 (n = 21) for HT×HT, and 6±SEM of 0.43 (n = 25) for HM×HM. The average number of pups in the infected group was 5.875±SEM of 0.76 (n = 9) (Figure 4C). When comparing the average number of normally born pups, infection with SASS-CoV-2 did not induce significant differences in pregnancy rates. To test the maternal–fetal trans-vertical infection of SARS-CoV-2, RT-PCR was performed using a primer set amplifying CoV_NP. Nucleocapsid RNA of the virus was detected only in the lungs of infected mice but not in the amnion, placenta, umbilical cord, or fetus (F1, F2, and F3). Compared to the lungs of non-infected control mice, severe lesions including the decrease of pulmonary alveoli were examined from the lungs of infected mice (Figure 4F). These data suggest that SARS-CoV-2 did not induce maternal–fetal trans-vertical infection, and the viral infection during the post-implantation stage did not affect embryonic development.

## DISCUSSION

As the aftermath of the COVID-19 pandemic continues, many controversial studies have been published regarding the vertical transmission of SARS-CoV-2. In humans, studies have suggested possible SARS-CoV-2 infection in the placenta and fetus. However, there is no experimental evidence to prove SARS-CoV-2 vertical transmission from the mother to her fetus. Moreso, the effect of SARS-CoV-2 infection on the female reproductive system is still unclear. Currently, the transgenic mouse model expressing hACE2 is widely used as an animal model for SARS-CoV-2 treatment or vaccination. However, the effect of infection on pregnancy outcomes in female mice has not been fully determined. In this study, we examined the effects of the viral infection on pregnancy in K18-hACE2 transgenic mice. K18-hACE2 transgenic mice are the most sensitive to SARS-CoV-2 and can model non-severe and severe SARS-CoV-2 affected patients in many animal models [25]. Studies using K18-hACE2 transgenic mice revealed the expression of hACE2 mRNA in the lungs, brain, kidneys, and small intestine [25,26]. In addition, it has been discovered that the neuro-invasion of SARS-CoV-2 is dependent on hACE2 expression [27]. In this study, we confirmed that hACE2 mRNA is expressed in the female reproductive system, including the uterus and ovaries. Furthermore, we used K18-hACE2 transgenic mice as an appropriate animal model to test the effect of SARS-CoV-2 infection on pregnancy.

According to the studies reported thus far, there are contradictory results related to the SARS-CoV-2 infection and pregnancy. Gao [28] showed that SARS-CoV-2 was not detected in the placenta or infants of eight affected pregnant women. Penfield et al [29] and Cribiù et al [30] reported the detection of SARS-CoV-2 in affected pregnant women's placentas but not in the fetus. In addition, Hosier et al [15] reported the detection of SARS-CoV-2 in the placenta and umbilical cord, which was localized predominantly in the syncytiotrophoblast of the maternal–fetal interface. Vivanti et al [31] reported that SARS-CoV-2 was identified in the placentas, amniotic fluid, and nasopharyngeal swabs of newborns on days 1, 3, and 18 after birth. Alzamora et al [32] also stated that SARS-CoV-2 was found in nasopharyngeal swabs of neonates 16 and 48 h after birth. In summary, these results can be categorized into three cases as follows: i) SARS-CoV-2 cannot infect the placenta; ii) SARS-CoV-2 can infect the placenta only; and iii) SARS-CoV-2 can infect the fetus through the placenta and umbilical cord. Almost all of the experiments mentioned above were conducted with patients with severe SARS-CoV-2 infection; hence, childbirth was through cesarean section. In humans, if cesarean section is performed, the placenta, umbilical cord, and fetus are highly susceptible to exposure to maternal blood [33]. For this reason, contamination of sampled human tissues cannot be completely excluded; therefore, the results can be controversial. However, in this study, tissue sampling was performed after sacrificing the mice. Thus, contamination through maternal blood to the reproductive system could be minimized.

In humans, ACE2- and TMPRSS2-positive cells have been identified in all three trimesters, trophectoderm, and placenta [34]. In addition, the expression of ACE2 and TMPRSS2 was confirmed in trophoblasts, blastocysts, syncytiotrophoblasts, and hypoblasts during the implantation stage [35]. This suggests that intrauterine infection with SARS-CoV-2 may directly affect the embryo and fetus through vertical transmission. The zygote, 2- and 4-cell embryos, morula, and blastocyst established by *in vitro* fertilization in K18-hACE2 homozygous female and male mice did not express hACE2 (no results). However, hACE2 mRNA expression was detected in the cumulus-oocyte complex, indicating the expression of hACE2 in cumulus cells. Given that in this study, viral infection was done after 2.5 dpc in which embryos without cumulus cells existed, the effect of SARS-CoV-2 infection on oocyte maturation and ovulation could not be tested. Therefore, the effect of a SARS-CoV-2 infection on the maturity and development of oocytes requires further investigation.

According to our results, a SARS-CoV-2 infection during the pre-implantation stage did not affect embryo implantation. A mouse model using hACE2-Chimera mice made with the CRISPR/Cas9 system showed reduced pregnancy rates in mice infected before E4.5. However, there were no significant differences in embryo resorption and embryo development after implantation between infected and uninfected mice [36]. Our data showing no detectable SARS-CoV-2 RNA in the uterus and ovaries suggest that intrauterine infection did not occur. In addition, this indicates that systemic inflammation through respiratory infection with SARS-CoV-2 cannot induce implantation failure or embryo resorption in the reproductive system.

Viral infection after normal implantation can interfere with embryonic development through modifications of the immune system, supporting fetal development, or direct viral infection of the uterus and placenta [13,14,37]. HSV induces the production of interferon gamma in the maternal decidua and placenta through innate immune responses and triggers tropism of fetal neural cells and inflammatory reactions in the brain [38]. ZIKV induces systemic inflammation through the expression of interleukin (IL)-1, IL-2, IL-6, IL-7, IL-15, and IL-16 and causes high levels of expression of inflammation-related chemokines and cytokines in the fetus [38,39]. Similarly, the direct interaction of human immunodeficiency virus with maternal neutralizing antibodies and placental macrophages causes damage to the placenta [38,39]. In particular, ZIKV infection triggers pathogenesis, such as trophoblast infection, apoptosis, vascular damage, hemorrhage, and loss of placental structure without direct fetal infection, resulting in fetal demise. Altogether, our data suggest that, unlike other viruses, SARS-CoV-2 cannot induce adverse pregnancy outcomes.

Infection with SARS-CoV-2 at the post-implantation stage did not induce a significant difference in pregnancy outcomes compared to the control group. This result is similar to previously published findings that state that embryo resorption did not occur in infected mice after E4.5 [26]. Viral RNA was not detected in the female reproductive tract, indicating that the virus cannot infect the reproductive tract or the fetus. In humans, the virus has been detected in both the placenta and fetus [29–32]. However, we could not identify viral mRNA in the female reproductive system of the infected K18-hACE2 transgenic mice. After viral infection, K18-hACE2 mice died at 6 to 7 dpc, so the effect of the viral infection on the first- and second-trimester stages could not be examined. Further studies should be conducted to confirm the effect of SARS-CoV-2 infection on the early stages of pregnancy after implantation in other animal models.

## Figures and Tables

**Table 1:** Primer sequences and real-time polymerase chain reaction conditions

Genes	Accession No.	Primer sequence	Annealing temperature (°C)	Product size (bp)
hACE2	NM_001371415.1	Fwd; 5′-AGGGCAAAGTTGATGAATGC-3′	59	208
		Rev; 5′-ACTTCTCGGCCTCCTTGAAT-3′		
*mACE2*	NM_001130513.1	Fwd; 5′-CTACAGGCCCTTCAGCAAAG-3′	59	204
		Rev; 5′-TGCCCAGAGCCTAGAGTTGT-3′		
*CoV_NP*	NC_045512.2	Fwd; 5′-ACCGAAGAGCTACCAGACGA-3′	60	246
		Rev; 5′-TTGGCAATGTTGTTCCTTGA-3′		
*GAPDH*	NM_001289726	Fwd; 5′-GGTGAAGGTCGGTGTGAACG-3′	59	233
		Rev; 5′-CTCGCTCCTGGAAGATGGTG-3′		

Fwd, forward primer; Rev, reverse primer.

hACE2, human angiotensin converting enzyme 2; *mACE2*, mouse angiotensin converting enzyme 2; *CoV_NP*, SARS-CoV-2 nucleocapsid; *GAPDH*, glyceraldehyde 3-phosphate dehydrogenase.
